# Beyond Sentinel Lymph Node: Outcomes of Indocyanine Green-Guided Pelvic Lymphadenectomy in Endometrial and Cervical Cancer

**DOI:** 10.3390/ijerph20043476

**Published:** 2023-02-16

**Authors:** Benito Chiofalo, Antonio Simone Laganà, Fabio Ghezzi, Camilla Certelli, Jvan Casarin, Valentina Bruno, Isabella Sperduti, Vito Chiantera, Panagiotis Peitsidis, Enrico Vizza

**Affiliations:** 1 Gynecologic Oncology Unit, Department of Experimental Clinical Oncology, IRCCS “Regina Elena” National Cancer Institute, 00144 Rome, Italy; 2 Unit of Gynecologic Oncology, ARNAS “Civico-Di Cristina-Benfratelli”, Department of Health Promotion, Mother and Child Care, Internal Medicine and Medical Specialties (PROMISE), University of Palermo, 90127 Palermo, Italy; 3 Department of Obstetrics and Gynecology, “Filippo Del Ponte” Women and Children Hospital, University of Insubria, 21100 Varese, Italy; 4 Biostatistical Unit, IRCCS “Regina Elena” National Cancer Institute, 00144 Rome, Italy; 5 Department of Obstetrics and Gynecology, Helena Venizelou Hospital, 115 21 Athens, Greece

**Keywords:** gynecologic oncology, minimally invasive surgery, indocyanine green, endometrial cancer, cervical cancer, robotic surgery, laparoscopy, pelvic lymphadenectomy

## Abstract

Background: The aim of our study was to compare the number of lymph nodes removed during indocyanine green (ICG)-guided laparoscopic/robotic pelvic lymphadenectomy with standard systematic lymphadenectomy in endometrial cancer (EC) and cervical cancer (CC). Methods: This is a multicenter retrospective comparative study (Clinical Trial ID: NCT04246580; updated on 31 January 2023). Women affected by EC and CC who underwent laparoscopic/robotic systematic pelvic lymphadenectomy, with (cases) or without (controls) the use of ICG tracer injection within the uterine cervix, were included in the study. Results: The two groups were homogeneous for age (*p* = 0.08), Body Mass Index, International Federation of Gynaecology and Obstetrics (FIGO) stages (*p* = 0.41 for EC; *p* = 0.17 for CC), median estimated blood loss (*p* = 0.76), median operative time (*p* = 0.59), and perioperative complications (*p* = 0.66). Nevertheless, the number of lymph nodes retrieved during surgery was significantly higher (*p* = 0.005) in the ICG group (*n* = 18) compared with controls (*n* = 16). Conclusions: The accurate and precise dissection achieved with the use of the ICG-guided procedure was associated with a higher number of lymph nodes removed in the case of systematic pelvic lymphadenectomy for EC and CC.

## 1. Introduction

Lymph node involvement is one of the most important prognostic factors in endometrial (EC) and cervical cancer (CC) [1,2]. Indeed, the presence of metastatic lymph nodes modifies the International Federation of Gynecology and Obstetrics (FIGO) stage for both tumors and plays a pivotal role impacting the post-surgical treatments [3,4]. As a consequence, retroperitoneal staging is required for the correct treatment of both EC and CC, and systematic pelvic lymphadenectomy still performed in different settings [5,6,7]. The sentinel lymph node (SLN) biopsy is a standard practice in breast cancer [8] and melanoma [9], and in recent years it has been used in some early-stage gynecological cancers, such as EC and CC, as an alternative to lymphadenectomy [10,11,12].

Indocyanine green (ICG) has been demonstrated to have high accuracy for the detection of SLN in EC and CC, especially in laparoendoscopic setting [12,13,14,15]. ICG is safe, cheap, and might also be helpful to obtain an accurate visualization of the lymphatic drainage during systematic pelvic lymphadenectomy and to guide the surgeon during the procedure [16,17]. Several studies have demonstrated an advantage of the ICG-guided lymphadenectomy in other types of cancer, by showing a higher number of lymph nodes removed with this technique when compared to the standard systematic lymphadenectomy (without ICG) [18,19,20]. As far as we know, there are no published studies about ICG-guided systematic pelvic lymphadenectomy in EC and CC. In this scenario, the aim of our study was to compare the number of lymph nodes removed by performing an ICG-guided pelvic lymphadenectomy versus the standard systematic pelvic lymphadenectomy in EC and CC.

## 2. Materials and Methods

### 2.1. Patients

We conducted a multicenter retrospective comparative study, collecting data from January 2014 to November 2018 (Clinical Trial ID: NCT04246580; updated on 31 January 2023). All the consecutive patients with apparent early-stage EC and CC, without suspicious radiologic appearance of lymph nodal metastasis, who underwent laparoscopic or robotic systematic bilateral pelvic lymphadenectomy were included. The minimally invasive approach was chosen also in case of CC, since until November 2018 we had no strong evidence showing poorer survival outcomes with this type of surgery compared to the open surgical approach [21]. Indeed, until 2018, the laparoscopic approach was a potential alternative to laparotomic surgery because it was associated with a lower morbidity rate and similar oncologic outcomes [22,23,24]. Nevertheless, since 2018, much has changed in the surgical concepts of cervical and uterine cancer, including indications of minimally invasive surgery, sentinel node biopsy, and pelvic lymph node dissection, as further discussed in next sections.

Pre-operative work-up included medical history collection, physical and vaginal-pelvic examination, chest X-ray, ultrasound scans and pelvic magnetic resonance imagining (MRI) in all patients and positron emission tomography/computed tomography (PET/CT) scans in CC patients, following an algorithm previously described [25]. Patients who underwent incomplete pelvic lymphadenectomy (SLN biopsy, lymph nodes sampling) were excluded from the current analysis. All the surgical reports were analyzed to select patients who underwent ICG-guided systematic pelvic lymphadenectomy (ICG-LND) and patients who underwent standard systematic pelvic lymphadenectomy (conventional-LND). The choice to perform ICG-LND or conventional-LND was not influenced by any clinical parameter and was based on surgeon’s preference. All the histological reports were analyzed and data about the number of lymph nodes removed during surgery (primary outcome) were recorded. Furthermore, data about the type of tumor (EC or CC) and FIGO stage were collected.

The study was approved by the Institutional Review Boards of the referral center (“Regina Elena” National Cancer Institute of Rome—#CE RS1285/19(2297)) and of the participating centers. The design, analysis, interpretation of data, drafting, and revisions are in compliance with the Helsinki Declaration, the Committee on Publication Ethics guidelines, and the Strengthening the Reporting of Observational Studies in Epidemiology (STROBE) Statement [26], validated by the Enhancing the Quality and Transparency of Health Research (EQUATOR) Network. The study was not advertised, and no remuneration was offered to encourage patients to give consent for collection and analysis of their data. Each patient enrolled in this study was informed about the aims and procedures and provided their informed consent to allow data collection for research purposes.

### 2.2. Indocyanine Infiltration Technique and Surgical Procedure

Briefly, 25 mg of ICG were diluted in 20 cc of sterile water in order to obtain a concentration of 1.25 mg/mL. Just before starting surgery, we performed a slow superficial (1–3 mm) and deep (1–2 cm) infiltration of 1 cc of ICG solution at 3 and 9 o’clock positions on the cervix with a spinal needle (20 G—90 mm). With this technique, the ICG was injected in the submucosal space and in the cervical stroma, near the blood vessels, an area with a rich lymphatic drainage. To see the diffusion of ICG, we used specific laparoscopic equipment: an optic with an infrared filter, a light source, and a high-definition camera. The diffusion of ICG can be divided in three phases: the first, which lasts a few seconds and shows the site of inoculation and the parametrium; the second (20–30 min later), in which the lymphatic system draining the site of inoculum is visible; and the third (1–2 h later), called the vascular phase, when the ICG goes in the vascular circulation, with a partial contamination of the surgical field. The best phase to perform the lymphadenectomy is the second one.

All patients underwent pelvic lymphadenectomy (with or without ICG), with complete removal of external and internal iliac lymph nodes up to the level of common iliac bifurcation and obturator lymph nodes. During procedures with ICG, a careful identification and isolation of lymphatic vessels and mapped lymph nodes was performed to avoid the extravasation of the ICG.

### 2.3. Statistical Analysis

Descriptive statistics were performed on characteristics of patients. The Chi-square, Fisher exact, and Mann–Whitney U-tests were used when comparing categories against categorical and continuous data, respectively. A *p*-value < 0.05 was considered statistically significant. SPSS software (SPSS version 21.0, SPSS Inc., Chicago, IL, USA) was used for all statistical evaluations.

## 3. Results

We included 230 patients: 61 who underwent ICG-guided systematic lymphadenectomy (ICG-LND group) and 169 who underwent conventional systematic lymphadenectomy without ICG injection (conventional-LND group). Patients’ clinicopathological characteristics are shown in Table 1.

The median age was 56 (range 30–77) and 58.3 (range 27–80) in the ICG group and in the conventional-LND group, respectively. There were no significant differences in patient age (*p* = 0.08), Body Mass Index (*p* = 0.52) (Table 1). The number of patients with lymph node metastasis (16.4% in the ICG group vs. 14.7% in conventional-LND group) and the number of metastatic lymph nodes detected (*p* = 0.14) were similar in the two groups.

The distribution of EC and CC was not homogeneous in the two groups (*p* = 0.01): in the conventional-LND group the percentage of EC was predominant, although in the ICG-LND group there was an equal distribution of the two types (Table 1). There were no significant differences in FIGO stages between the two groups, both in EC (*p* = 0.41; Table 2) and in CC (*p* = 0.17; Table 3) patients.

**Figure 1:**
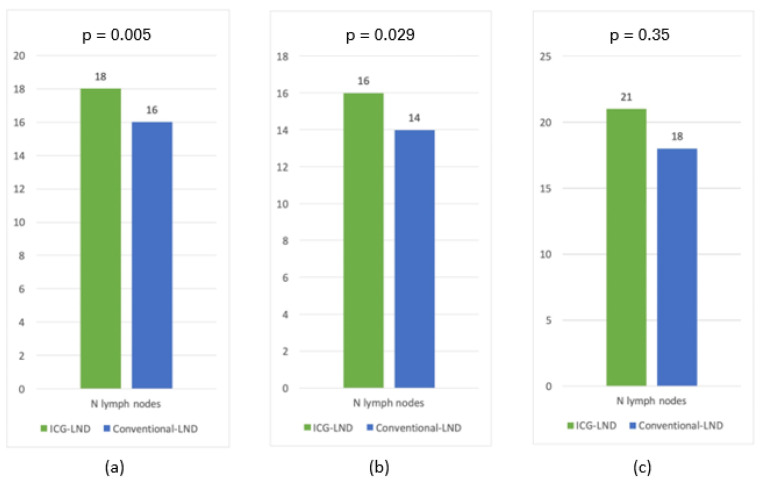
Median number of harvest lymph nodes in patients who underwent either ICG guided systematic pelvic lymphadenectomy (ICG-LND) or standard systematic pelvic lymphadenectomy without ICG (Conventional-LND): (**a**) overall (endometrial and cervical cancer); (**b**) endometrial cancer; (**c**) cervical cancer.

We did not find significant differences regarding the operative time (*p* = 0.59), estimated blood loss (*p* = 0.76), or peri-operative complications (*p* = 0.67) between the two groups (Table 4). When the number of lymph nodes retrieved during surgery was compared, it was significantly higher (*p* = 0.005) in the ICG-LND group (Table 4; Figure 1a).

In particular, in EC patients, the median number of lymph nodes retrieved was 16 and 14 (Figure 1b) in the ICG-LND group and in the conventional-LND group (*p* = 0.029), respectively.

In CC patients, the median number of lymph nodes retrieved was 21 and 18 (Figure 1c) in the ICG-LND group and in the conventional-LND group (*p* = 0.35), respectively.

## 4. Discussion

Our data analysis showed that in patients surgically treated for apparent early-stage EC, the injection of ICG increases the number of lymph nodes removed during systematic pelvic lymphadenectomy. Our results might be attributed to the optimal visualization of the lymphatic drainage achieved following the tracer migration. In early-stage CC, more lymph nodes were achieved in the ICG-LND group, however the difference between the two groups was not statistically significant.

ICG was commonly used in the SLN technique in different gynecological cancers [10,11], and evidence is further accumulating also for early-stage ovarian cancer [27,28]. Even in the recent guidelines by the National Comprehensive Cancer Network [29], SLN biopsy is considered in the management of EC confined to the uterus and without suspicious lymph node involvement at preoperative imaging. In CC, the indications for SLN biopsy are FIGO stage IA1 with lymphovascular space invasion, IA2, IB1 and IIA1 tumors [29]. A high detection rate (95%) has been demonstrated for tumors smaller than 2 cm (sensitivity of 100%) and the SLN technique is contraindicated in the case of extra-cervical invasion [30,31].

Although the SLN biopsy has the advantage of a potential reduction of postoperative complications compared with a more invasive surgical procedure, in advanced and aggressive diseases the systematic pelvic lymphadenectomy remains the best choice. In these cases, ICG may maintain a role by improving the surgical technique and by showing the presence of anomalies in the lymphatic drainage [32]. In this scenario, our study showed a higher number of pelvic lymph nodes removed during surgery for both EC or CC by using ICG-guided lymphadenectomy, confirming what has been already found for other non-gynecologic cancers [18,19,20]. Indeed, some authors analyzed the ICG-guided lymphadenectomy during robotic radical gastrectomy for gastric cancer and showed the absence of procedure-related complications [20]. Kim et al. reported the ICG-guided central node dissection during robotic thyroidectomy [18]. Other authors evaluated this technique for the surgical treatment of medium-low rectal cancer, and showed even a reduction in blood loss [19]. Regarding pelvic lymphadenectomy, some authors showed that the use of ICG can improve the quality of extended pelvic node dissection in patient undergoing prostatectomy for prostate cancer [33]. Our study did not show significant differences in blood losses and peri-operative complications between the ICG group and conventional LND. Otherwise, focusing only on vascular accidents, which are complications directly related to lymphadenectomy, we reported the absence of this complication just in the ICG group. These limited data may suggest that ICG could reduce the risk of vascular complications, by improving the differentiation between the lymphatic tissues and the surrounding anatomical structures, but largest prospective trials are required to confirm this hypothesis. Taking together all the pieces of available evidence, not only the ICG injection has been found to improve the quality of lymphadenectomy, but it may also have further applications, which have been reported in the literature: ICG has been used to improve the surgical outcomes in the type C1 radical hysterectomy and in the total mesorectal and mesocolic excision [34,35,36]. Furthermore, it can be used for the visualization of important structures such as the ureters, in order to reduce the risk surgical lesions. In addition, Kimming et al. suggested the use of ICG during aortic lymphadenectomy to save nervous structures, that can be mistaken for lymphatic vessels [37]. Finally, some studies have suggested an advantage of ICG injection even in the retrieval of lymph nodes from the resected specimen for pathologists or in shortening the learning curve for surgeons who are not familiar with lymph nodal surgery [19,20].

The ICG injection with all its applications may improve the pelvic lymphadenectomy by allowing a visualization of each draining lymph node. Even in obese patients with a high amount of fat and soft tissue, lymph nodes detection may be simplified by this technique [20]. In addition, the complete visualization of the lymphatic vessels and lymph nodes may avoid the breakage of fragile lymphatic structures with cells spillage and the lesion of blood vessels and nervous structures [20,38]. Although our retrospective analysis does not allow to draw firm conclusions, probably some lymph nodes can escape the surgeon’s eye with conventional-LND and, in this context, ICG may help to distinguish lymphatic structures from the visceral fat.

According to our results, the ICG-guided lymphadenectomy did not increase the operation time, and the ICG infiltration is an easy and cheap way to perform this surgery. The most important weaknesses of our study are the retrospective observational setting and the small cohort of patients, which were not randomized to receive ICG-guided or conventional lymphadenectomy. Due to the low number of enrolled women, it was not possible to perform a multivariate analysis to determine the impact of some possible confounding factors, such as type of surgery and BMI, but both groups were homogeneous for these parameters.

Although we used a minimally invasive for both EC and CC, we acknowledge that the publication of the Laparoscopic Approach to Cervical Cancer Trial (LACC) trial in November 2018 led to a profound paradigm shift in the surgical management of CC [21].

Indeed, according to the new evidence and for reasons not yet fully explained despite countless opinions, the use of minimally invasive surgery for CC, even when performed in centers with high volume and proven surgical expertise for gynecologic malignancies, was associated with higher rates of recurrence and mortality compared with open approach. A similar increase in mortality and recurrence rates of laparoscopic surgery compared with laparotomic surgery was also found in another American study, based on a large case series [39]. Following these data analyses, all the advantages in terms of morbidity offered by the laparoscopic approach are then side-stepped considering the worse oncologic outcomes compared to radical abdominal hysterectomy, forcing the surgeon to “come back” to an open approach for the surgical management of CC.

The question therefore arose as to whether there were factors that could justify the superiority of radical abdominal hysterectomy. A large multicenter retrospective analysis and other studies that followed from it [40,41] supported a potential impact of tumor spillage and macroscopic tumor manipulation at the time of surgery on the increased risk of recurrence associated with the minimally invasive technique. Interestingly, patients who did not undergo conization preoperatively, and therefore had cancer present at the time of surgery, had a higher risk of recurrence after surgery than patients who underwent conization prior to hysterectomy [42]. This could (at least in part) support the hypothesis that the use of carbon dioxide may play a deleterious role in oncological outcomes by increasing the risk of abdominal implants [43,44].

The results of the LACC trial also have important implications for the education and training of young gynecological surgeons and residents [45]. Lewiki et al. [46] showed that since the publication of the LACC trial, the minimally invasive approach for cervical neoplasms has been adopted less and less, even in academic centers. Considering that studies on large case series have shown that proper minimally invasive surgical training can led gynecological surgeons to obtain similar oncological outcomes by both minimally invasive and open surgeons with almost same proficiency [47], the abandonment of these kinds of techniques in university hospitals could be a problem that may be difficult to escape from. Teaching future surgeons the correct minimally invasive approach for gynecological neoplasms, including for CC, could help to identify the correct time on their learning curve to leave them alone to operate on this kind of tumor. Therefore, it would be advisable to design trials analyzing only oncological outcomes from teams with proven experience and undergoing proper training for minimally invasive surgery [45]. The more experienced gynecological surgeons should therefore teach minimally invasive techniques even for the management of CC as well, so that if new evidence leads to a new minimally invasive surgical indication in the future (for instance from the ongoing “Robot-assisted approach to cervical cancer” international multi-center, open-label randomized controlled trial [48]), there will be still adequate surgical proficiency to treat these patients.

Interestingly, since the publication of the LACC trial, radical hysterectomy by vaginal approach, which had been sidelined for years, has regained timely application in CC surgery [49,50,51,52,53]. The development of a vaginal manchette before performing laparoscopic radical hysterectomy may be an appropriate protective maneuver to avoid tumor spillage and potentially justify the minimally invasive approach by avoiding the negative outcomes of minimally invasive surgery in early-stage CC [54,54]. Actually, the LACC trial led to the development of new surgical approaches for CC, such as the combination of radical vaginal and laparoscopic surgery that could be a viable alternative to “pure” laparoscopy albeit in need of further investigation. Nevertheless, radical vaginal surgery and the creation of a vaginal manchette entail a specific learning curve and, although every gynecologic oncologist should be able to perform these techniques, to date they are not widely used and taught in many centers (even academic ones).

A possible alternative to laparoscopic surgery could also be robotic surgery [55,56,57,58]. However, current evidence on this approach is not robust enough to draw a firm conclusion, and trials on this issue should be encouraged.

Despite the impact that the LACC trial has had on the scientific community, some studies conducted before this study did not report the same results in terms of mortality and recurrence, recording all the benefits of the minimally invasive approach and reporting a similar number of lymph nodes recovered following systematic pelvic and/or aortic lymphadenectomy comparing laparoscopy and open surgery [59].

Therefore, laparoscopic radical hysterectomy, although technologically more attractive, is no longer an option for patients with CC and, if a minimally invasive approach is proposed, patients must be informed in detail about the current scientific debate and available data, although future evidence may change the current indications [5,60].

Anyway, the overall scope of this study was not to suggest an alternative procedure to sentinel node biopsy, which remains the best procedure for endometrial cancer and early-stage cervical cancer. Conversely, we aim to suggest the use of ICG to guide pelvic lymphadenectomy in the common surgical practice for gynecologic malignancies, especially when a systematic lymphadenectomy is still necessary (for instance, in the case of bulky lymph nodes and FIGO IB3-IIA1 cervical cancer) [61,62].

To the best of our knowledge, this is the first study that analyzed ICG-guided pelvic systematic lymphadenectomy in EC and CC. On that basis, we take the opportunity to solicit future randomized controlled trials with a larger cohort to confirm our preliminary results.

## 5. Conclusions

The use of ICG-guided pelvic lymphadenectomy for EC or CC may allow, on the one hand, to be more radical by increasing the number of lymph nodes removed and, on the other hand, to be more accurate and precise for the dissection of draining lymph nodes.

## Figures and Tables

**Table 1:** Clinical characteristics of the investigated population.

	ICG-LND ^a^	Conventional LND ^b^	*p*
Total	61	169	
Median age	56 (30–77)	58.3 (27–80)	0.08
Median BMI	26 (21–39)	25 (18–56)	0.52
Type of cancer			0.01
Endometrial cancer	33 (54.1%)	122 (72.2%)	
Cervical cancer	28 (45.9%)	47 (27.8%)	
Patients with lymph node metastasis	10 (16.4%)	25 (14.7%)	0.76

Data are shown as median (range) or *n* (%). BMI: body mass index. ^a^ ICG-guided systematic pelvic lymphadenectomy. ^b^ Standard systematic pelvic lymphadenectomy (without ICG).

**Table 2:** Final stage in women affected by endometrial cancer.

	ICG-LND ^a^	Conventional LND ^b^	*p*
Total	33	122	
FIGO stage			0.41
IA	15 (45.5%)	44 (36.1%)	
IB	13 (39.4%)	37 (30.3%)	
II	2 (6.1%)	14 (11.5%)	
IIIA	0	6 (4.9%)	
IIIB	0	3 (2.5%)	
IIIC	3 (9%)	18 (14.8%)	

Data are shown as *n* (%). FIGO: International Federation of Gynecology and Obstetrics. ^a^ ICG-guided systematic pelvic lymphadenectomy. ^b^ Standard systematic pelvic lymphadenectomy (without ICG).

**Table 3:** Final stage in women affected by cervical cancer.

	ICG-LND ^a^	Conventional LND ^b^	*p*
Total	28	47	
FIGO stage			0.17
IA1	2 (7.1%)	5 (10.6%)	
IA2	0	5 (10.6%)	
IB1	23 (82.1%)	27 (57.4%)	
IB2	1 (3.6%)	2 (4.3%)	
IIA1	2 (7.1%)	3 (6.4%)	
IIA2	0	0	
IIB	0	5 (10.6%)	

Data are shown as *n* (%). FIGO: International Federation of Gynecology and Obstetrics. ^a^ ICG-guided systematic pelvic lymphadenectomy. ^b^ Standard systematic pelvic lymphadenectomy (without ICG).

**Table 4:** Perioperative parameters.

	ICG-LND ^a^	Conventional LND ^b^	*p*
Total	61	169	
Type of surgery			0.007
LPS	59 (96.7%)	140 (82.8%)	
ROB	2 (3.3%)	29 (17.2%)	
Harvested lymph nodes (median)	18 (8–42)	16 (2–45)	0.005
Perioperative complications	3 (4.9%)	23 (13.6%)	0.66
Vascular complications	0 (0%)	4 (2.3%)	0.58
Median operative time (min)	140 (70–380)	140 (45–385)	0.59
Median estimated blood loss (mL)	75 (20–600)	100 (5–1000)	0.76

Date are expressed as median or *n* (%), as appropriate. LPS: laparoscopic surgery; ROB: robotic surgery. ^a^ ICG-guided systematic pelvic lymphadenectomy. ^b^ Standard systematic pelvic lymphadenectomy (without ICG).

## Data Availability

Anonymized dataset of the study will be available for the first author (B.C.) on reasonable request.
